# Surgery improves the prognosis of colon mucinous adenocarcinoma with liver metastases: a SEER-based study

**DOI:** 10.1186/s12885-020-07400-4

**Published:** 2020-09-23

**Authors:** Jia Huang, Guodong Chen, Huan Liu, Yiwei Zhang, Rong Tang, Qiulin Huang, Kai Fu, Xiuda Peng, Shuai Xiao

**Affiliations:** 1 Institute of Clinical Medicine of the First Affiliated Hospital, University of South China, Hengyang, Hunan 421001 People’s Republic of China; 2 Hengyang Medical College, University of South China, Hengyang, Hunan 421001 People’s Republic of China; 3 Department of Surgery of the First Affiliated Hospital, University of South China, Hengyang, Hunan 421001 People’s Republic of China; 4 Department of Gastrointestinal Surgery of the First Affiliated Hospital, University of South China, Hengyang, Hunan 421001 People’s Republic of China; 5 Institute of Molecular Precision Medicine and Hunan Key Laboratory of Molecular Precision Medicine, and Department of General Surgery, Xiangya Hospital, Central South University, Changsha, Hunan 410008 People’s Republic of China; 6 Department of Surgery of the Second Affiliated Hospital, University of South China, Hengyang, Hunan 421001 People’s Republic of China

**Keywords:** Colon carcinoma, Mucinous adenocarcinoma, Liver metastases, Surgery, Survival

## Abstract

**Background:**

Mucinous adenocarcinoma (MC) is the second most common pathological type of colon carcinoma (CC). Colon cancer liver metastases (CLMs) are common and lethal, and complete resection of the primary tumour and metastases for CLM patients would be beneficial. However, there is still no consensus on the role of surgery for MC with liver metastases (M-CLM).

**Methods:**

Patients diagnosed with M-CLM or classical adenocarcinoma with CLM (A-CLM) from 2010 to 2013 in the Surveillance, Epidemiology, and End Results (SEER) database were retrieved. The clinicopathological features and overall survival (OS) and cancer-specific survival (CSS) data were compared and analysed.

**Results:**

The results showed that the M-CLM group had a larger tumour size, more right colon localizations, higher pT and pN stages, more female patients, and more retrieved and positive lymph nodes and accounted for a higher proportion of surgeries than the A-CLM group. The OS and CSS of M-CLM patients who underwent any type of surgery were significantly better than those of patients who did not undergo any surgery, but poorer than those of A-CLM patients who underwent surgery. Meanwhile, the OS and CSS of M-CLM and A-CLM patients who did not undergo any surgery were comparable. Compared with hemicolectomy, partial colectomy led to similar or better OS and CSS for M-CLM, and surgery was an independent protective factor for long-term survival in M-CLM.

**Conclusions:**

M-CLM had distinct clinicopathological characteristics from A-CLM, and surgery could improve the survival and is an independent favourable prognostic factor for M-CLM. In addition, partial colectomy might be a non-inferiority choice as hemicolectomy for M-CLM according to the results from this study.

## Background

Colon carcinoma (CC) is one of the most common and lethal cancers in the world [1]. A large proportion of CC deaths are due to metastasis, and more than 20% of patients have developed distant metastases by the time of diagnosis [2]. Although the mortality of all CCs is declining, the 5-year survival rate of metastatic CC (mCC) is still miserable and less than 10% [3]. The liver is the most frequent target organ for mCC, with liver metastasis (LMs) occurring in up to 25% of stage IV patients [4]. Complete resection of the primary tumours and metastatic lesions for some highly selected resectable colon cancer liver metastasis (CLM) patients is advocated by guidelines and provides better survival than non-surgical treatment, but less than 20% of this population meets the criteria for the procedures [5–7].

Mucinous adenocarcinoma (MC) is the second most common pathological type after classical adenocarcinoma (AC) among CCs and accounts for 10–15% of all CC patients [8]. According to the WHO, MC is defined as more than 50% of the lesion being composed of extracellular mucin. The molecular characteristics of MC are a relatively higher mutation rate of BRAF and KRAS, a greater proportion of the microsatellite instability high (MSI-H) and CpG island methylator phenotype, and greater expression of HATH1 and MUC2 than AC [9–11]. The pathogenesis of MC is poorly understood, and bacterial biofilms, inflammatory bowel diseases (IBDs) and radiotherapy are considered as potential risk factors [12, 13]. MC is frequently located in the proximal colon and has shorter survival and poorer systemic treatment response than AC, thus, MC is always suggested as a poor prognostic predictor for CC [9, 14–16]. Therefore, we should lend greater focus to the clinical management of MC patients.

To date, the prognosis of MC remains highly controversial, mainly because of the treatment strategy deviation for metastatic disease [8, 14, 17]. Although MC has a greater propensity for peritoneal dissemination than AC, the liver is still the most common metastatic site and accounts for up to 50% of all metastases [18, 19]. Management of these MC CLM (M-CLM) patients has long been controversial. One important reason is that M-CLM is frequently accompanied by metastases of other sites, thus, a large proportion of M-CLM tumours are traditionally considered unresectable unless emergency circumstances are present, and many studies suggest that incomplete resection is associated with high recurrence, poorer survival, and tumour growth and progression [10, 20–23]. However, the relatively poor response to chemotherapy of metastatic MC indicates that surgery may occupy a more important role in the treatment of these patients, although the probability of recurrence remains high [14, 24, 25]. Thus, some studies found that MC patients with complete resection of the primary lesion and M-CLM had poorer survival than AC CLM patients (A-CLM), but another study found that surgery for Union for International Cancer Control (UICC) stage IV MC could provide comparable survival to that of AC patients [8, 17, 19]. Furthermore, there is still no research investigating the role of surgery for M-CLM patients who cannot undergo radical resection. These situations and discrepancies highlight the need for more determine the role of surgery in the treatment of M-CLM.

In this study, we explored the prognosis of M-CLM patients who did or did not undergo surgery for the primary and metastatic lesions or both. The purpose of this study was to clarify the value of surgery and the prognostic factors for M-CLM patients from the Surveillance, Epidemiology, and End Results program (SEER 18, 1975–2016 varying).

## Methods

### Data source

The current study relied on the SEER cancer registry, which is a publicly available and reliable database and could provide follow-up information regarding the vital survival status and death causes. We required cases from 18 SEER registries with the anonymous data and obtained permission to download the research data file from the SEER database, which did not require further informed patient consent.

### Patients selection

We accessed the SEER database using the SEER program (www.seer.cancer.gov) and.

Surveillance Research Program, National Cancer Institute SEER*Stat software (www.seer.cancer.gov/seerstat) version 8.3.6, and obtained patients diagnosed with CLM between 2010 and 2013. The study included CLM patients according to the following criteria: 1) the International Classification of Disease for Oncology, Third Edition (ICD-O-3) site codes: cecum, ascending colon, hepatic flexure, transverse colon, splenic flexure, descending colon and sigmoid colon; 2) ICD-O-3 behavior codes: malignant; 3) diagnostic confirmation: positive histology; 4) ICD-O-3 histology codes: 8140/3: adenocarcinoma, NOS, 8480/3: mucinous adenocarcinoma; 5) American Joint Committee on Cancer (AJCC) 7th edition: M1a; 6) Vital status: alive, dead. The exclusion criteria were in the following: 1) surgery of primary site: blanks; 2) surgery of other regional site and distant site: blanks; 3) site-specific factor 1 (carcinoembryonic antigen, CEA): blanks; 4) age at diagnosis: unknown; 5): Total number of in situ/malignant tumours: unknown; 6) survival months: unknown; 7) other metastases site with this exception of liver metastasis.

The definition of partial colectomy (Code 30, SEER Program Code Manual, 3rd Edition) means the resection bowel with margins of about 10 cm which is less than hemicolectomy, such as ascending colon colectomy and transverse colon colectomy, but with adequate lymph node dissection. Hemicolectomy (code 40, SEER Program Code Manual, 3rd Edition) means right or left hemicolectomy or greater (but less than total colectomy), which means all of right or left colon and a portion of transverse are removed with adequate lymph node dissection.

### Outcome measures

For each patient, the survival outcomes were defined and analyzed: 1) overall survival (OS) was defined as the time from the date of diagnosis to death from any cause; 2) cancer-specific survival (CSS) was defined as the time from the date of diagnosis until cancer-associated death.

### Statistical analysis

Patient characteristics were summarized in descriptive statistics, and we compared differences in baseline characteristics between the M-CLM groups and A-CLM groups. Continuous data were compared using the one-way ANOVA test, and categorical variables were compared using the chi-square test. The Kaplan-Meier curves were used to estimate OS and CSS, and the log-rank test was used to compare the differences among groups. The prognostic factors associated with OS and CSS were analyzed by univariate and multivariable Cox proportional regression model, and then hazard ratios (HRs) and 95% confidence intervals (CIs) were estimated. All statistical analyses were performed with SPSS Statistical Package version 22.0 (SPSS Inc., Chicago, IL, USA), and *P* < 0.05 was considered to be statistical significant.

As a retrospective study based on SEER, there would be some confounding biases by inherent differences between demographic information. Thus, a one-to-one propensity-score matching (PSM) was employed to match the A-CLM and M-CLM groups using a logistic regression model based on the race, age and sex variables. Nearest neighbor matching was performed in a 1:1 ratio; A-CLM group was matched within its control M-CLM group. The caliper used for matching in this study was set at 0.001. The clinicopathological characteristics of the two groups were reevaluated after PSM (Table S1), as well as the follow-up status (Fig. S1).

## Results

### General demographic and clinicopathological characteristics of M-CLM

A total of 7179 patients were retrieved from the SEER database according to the inclusion and exclusion criteria. Then, according to the SEER Combined Metastasis at DX-liver (2010+) code, a total of 5816 CLM patients from 2010 to 2015 were enrolled, including 306 M-CLM patients and 5510 A-CLM patients. The results showed that M-CLM patients had the general features of MCs, such as larger tumour sizes, more localizations to the right colon, and higher pT and pN stages than A-CLM patients (*P* < 0.05 each, Table 1). In addition, the results also showed that the M-CLM group had more female patients and more retrieved and positive lymph nodes and accounted for a higher proportion of surgeries than the A-CLM group (*P* < 0.05 each, Table 1). Other variables, such as race, age, CEA level, number of primary tumours and tumour differentiation, were comparable between the two groups (*P* > 0.05 each, Table 1). In order to reduce the possible statistical biases, we performed 1:1 PSM analyis as described in methods and produced 306 patients in the A-CLM group and the M-CLM group respectively. Results showed that the clinicopathological characteristics and surgery information of the A-CLM and M-CLM group patients after PSM were strongly in line with the original data before PSM (Table S1), which strengthened the fingdings.

**Table 1:** The general demographic and clinicopathological features of mucinous colon adenocarcinoma liver metastasis (M-CLM) and classical colon adenocarcinoma liver metastasis (A-CLM) patients

Variables	A-CLM (5510)	M-CLM (306)	***P*** value
**Race**			
White	4102 (74.4%)	232 (75.8%)	
Black	935 (17.0%)	55 (18.0%)	
Others	473 (8.6%)	19 (6.2%)	0.501
**Age (years)**			
≤ 60	2023 (36.7%)	101 (33.0%)	
>60	3487 (63.3%)	205 (67.0%)	0.190
**Sex**			
Female	2439 (44.3%)	153 (50.0%)	
Male	3071 (55.7%)	153 (50.0%)	0.049
**CEA**			
Normal	593 (10.8%)	33 (10.8%)	
Elevated	3244 (58.9%)	184 (60.1%)	
Unknown	1673 (30.4%)	89 (29.1%)	0.890
**Primary tumor size (cm)**			
≤ 5	2408 (43.7%)	119 (38.9%)	
>5	1860 (33.8%)	156 (51.0%)	
Unknown	1242 (22.5%)	39 (10.1%)	< 0.001
**Tumor number**			
Solitary	4435 (80.5%)	248 (81.0%)	
Multiple	1075 (19.5%)	58 (19.0%)	0.811
**Location**			
Right colon	2403 (43.6%)	181 (59.2%)	
Transverse colon	486 (8.8%)	34 (11.1%)	
Left colon	2621 (47.6%)	91 (29.7%)	< 0.001
**Differentiation**			
Grade I/II	3634 (66.0%)	188 (61.4%)	
Grade III/IV	1172 (21.3%)	79 (25.8%)	
Unknown	704 (12.8%)	39 (12.7%)	0.158
**pT stage**			
**0–2**	562 (10.2%)	24 (7.8%)	
**3–4**	3892 (70.6%)	260 (85.0%)	
Unknown	1054 (19.1%)	22 (7.2%)	< 0.001
**pN stage**			
**N0**	1668 (30.3%)	62 (20.3%)	
**N+**	3391 (61.5%)	233 (76.1%)	
Unknown	451 (8.2%)	11 (3.6%)	< 0.001
**Examined lymph nodes**	12.52 ± 11.43	16.26 ± 10.41	< 0.001
**Positive lymph nodes**	4.41 ± 5.04	5.37 ± 5.84	0.003
**Surgery type**			
No surgery	1627 (29.5%)	34 (11.1%)	
Any surgery	3876 (70.3%)	272 (88.9%)	
Unknown	7 (0.2%)	0 (0%)	< 0.001

### Long-term survival in M-CLM

**Fig. 1:**
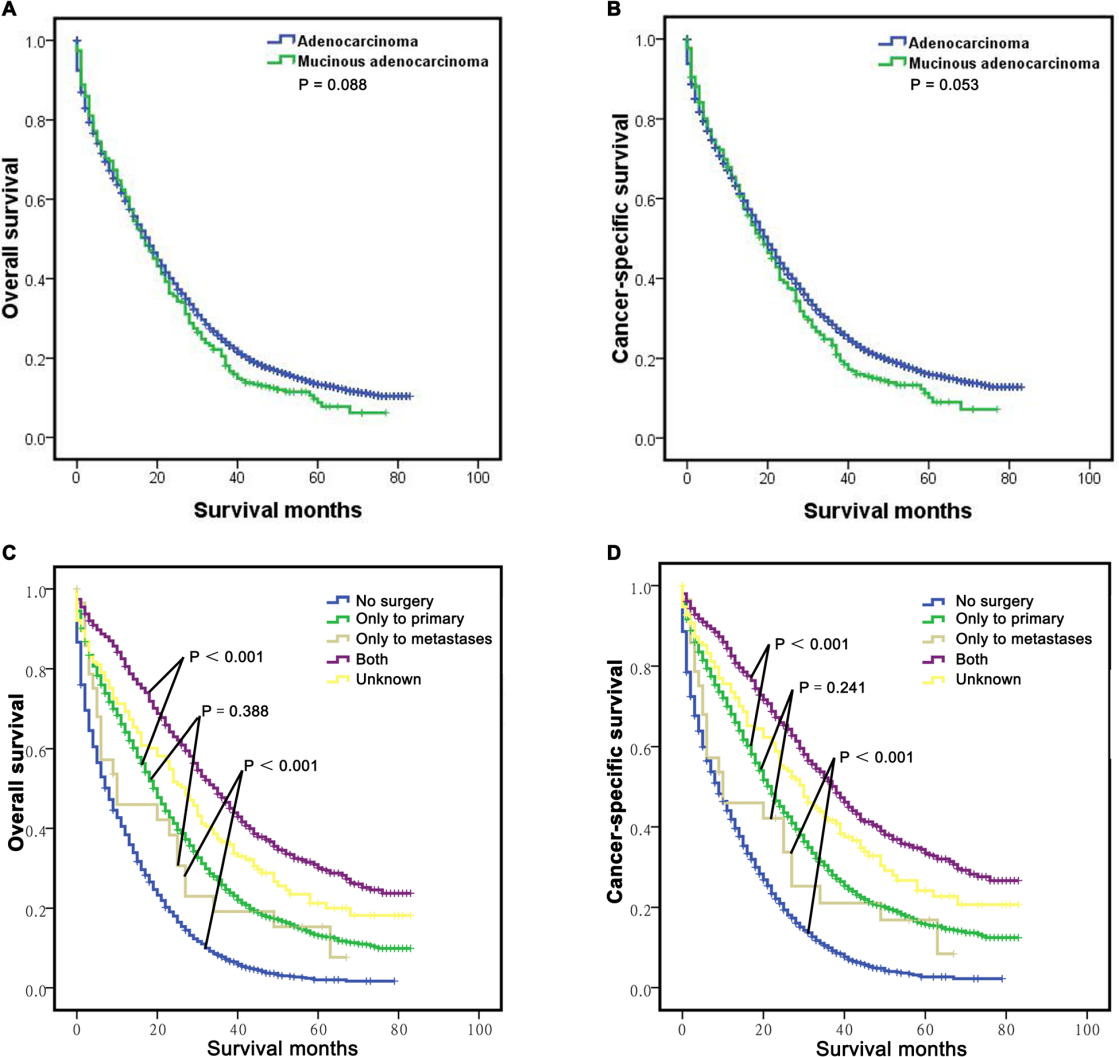
Long-term survival of CLM. A-B: The survival curves showed that the overall M-CLM group had similar overall survival (OS) **a** and cancer-specific survival (CSS) **b** with the A-CLM group; **c-d**: The survival curves showed that the CLM group who accepted the resection both to primary lesion and metastatic lesion had the best OS **c** and CSS **d**, followed by the resection only to primary lesion or metastatic lesion which had similar OS and CSS, and the patients who didn’t receive any surgery had the poorest OS and CSS

We then analysed the potential survival difference between M-CLM and A-CLM patients via Kaplan-Meier analysis and log-rank tests. The results showed that the follow-up of the whole study cohort was 0–83 months, and the median follow-up was 17.0 months. The OS of M-CLM patients was comparable to A-CLM patients (22.59 ± 1.24 vs. 25.65 ± 0.36 months, *P* = 0.088, Fig. 1a). The CSS of M-CLM patients was also similar to that of A-CLM patients (24.33 ± 1.33 vs. 28.19 ± 0.39 months, *P* = 0.053, Fig. 1b); although the actual values of the OS and CSS of M-CLM were lower than those of A-CLM, the difference was not statistically significant. The finding of OS and CSS of M-CLM patients were similar as A-CLM patients was also comfirmed after PSM (Fig. S1A, B).

### Long-term survival in M-CLM classified by surgery type

**Fig. 2:**
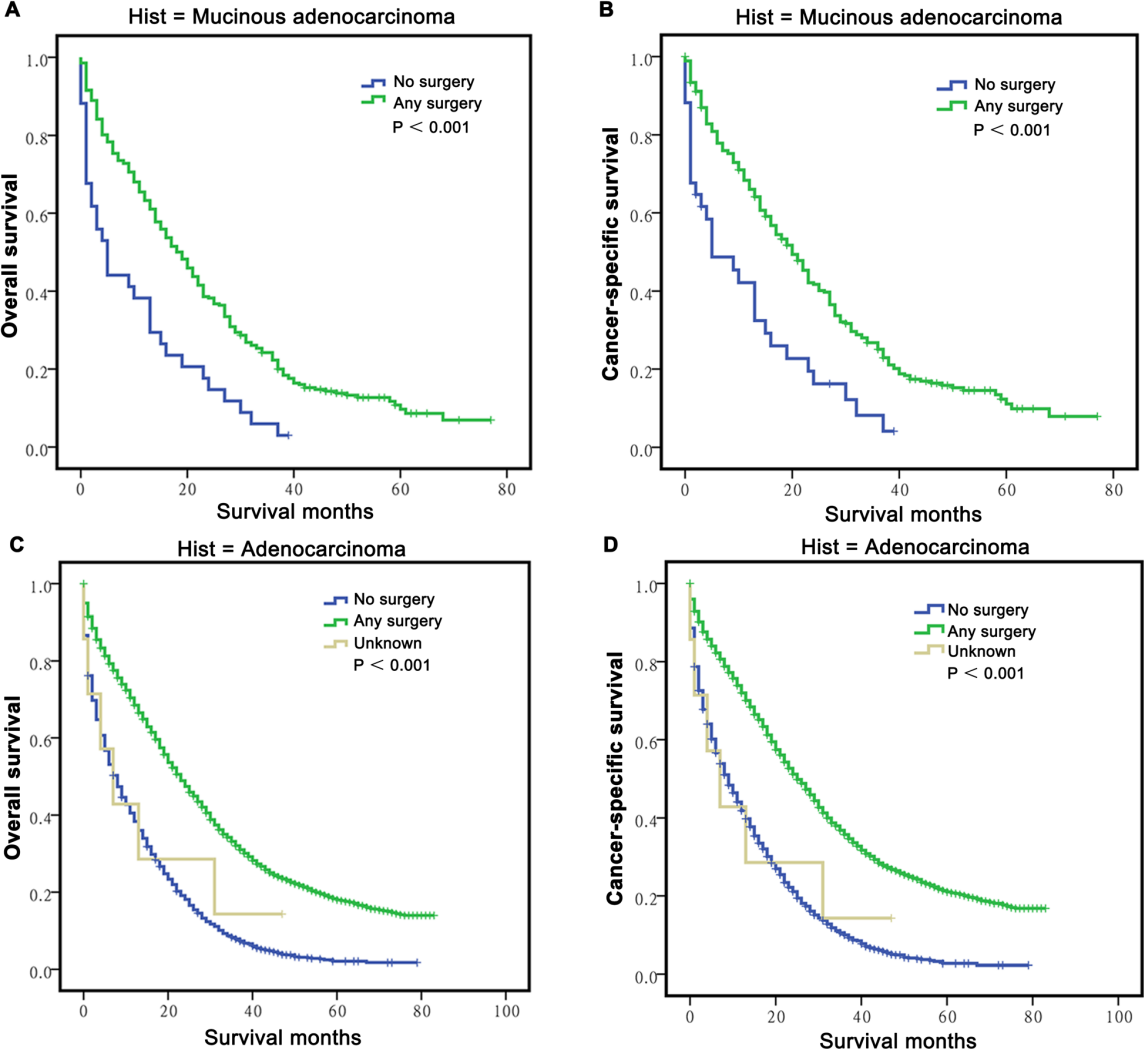
Long-term survival of CLM grouped by surgery and stratified by histology. A-B: The survival curves showed that the M-CLM patients who received any surgery had better OS **a** and CSS **b** than those didn’t accept any surgery; **c-d**: The survival curves showed A-CLM patients who received any surgery also had better OS **c** and CSS **d** than those didn’t accept any surgery

Furthermore, we explored the potential advantage of different surgery types for long-term survival. The results showed that the cohort who underwent resection for both the primary tumour and liver metastases had the best OS (41.15 ± 0.96 months, *P* < 0.001), followed those who underwent resection only for the primary lesion (26.79 ± 0.47 months) and for metastatic lesions (21.44 ± 4.22 months), which had similar OS (*P* = 0.388), and the patients who did not undergo any surgery had the poorest OS (13.08 ± 0.39 months, *P* < 0.001) (Fig. 1c). These results were also confirmed for the CSS analysis (Fig. 1d). Then, we classified and analysed the effect of surgery on the survival of M-CLM and A-CLM patients. The results showed that M-CLM patients who underwent any type of surgery (primary or metastatic lesion resection or both) had significantly better OS and CSS than those who did not undergo any type of surgery (*P* < 0.001 for all, Fig. 2a-b). The survival analyses in the A-CLM group also yielded similar results (*P* < 0.001, Fig. 2c-d).

### Survival differences between M-CLM and A-CLM stratified by surgery type

**Fig. 3:**
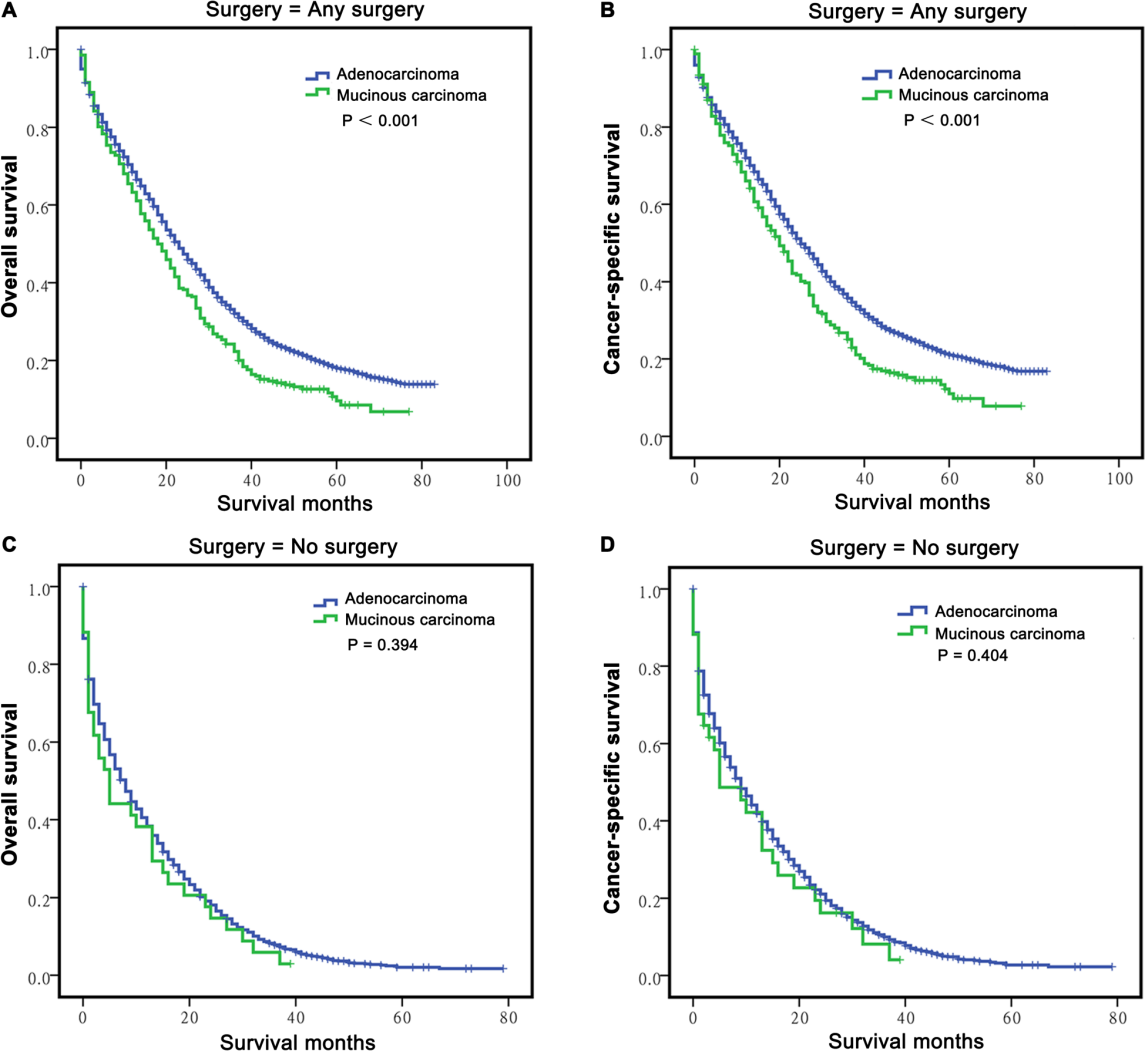
The stratified analysis for long-term survival of CLM according to surgery type. A-B: The survival curves showed that the M-CLM group received any surgery had poorer OS **a**) and CSS **b**) than the A-CLM group; **c-d**: the survival curves showed M-CLM and A-CLM groups had similar OS **c** and CSS **d** when didn’t perform any surgery

We previously found that M-CLM had comparable OS and CSS to A-CLM (Fig. 1A-B), since surgery could result in survival benefits for both cancers, and so we further analysed the potential survival differences between M-CLM and A-CLM via stratification of surgery types. The results showed that among all patients who underwent any kind of surgery, M-CLM patients had poorer OS (P < 0.001, Fig. 3a) and CSS (*P* < 0.001, Fig. 3b) than A-CLM patients. However, the OS and CSS were not significantly different between M-CLM and A-CLM patients who did not undergo surgery (*P* = 0.394 and *P* = 0.404, respectively, Fig. 3c-d). Kaplan–Meier OS and CSS curves after PSM also indicated the similar results (Fig. S1C-F).

**Fig. 4:**
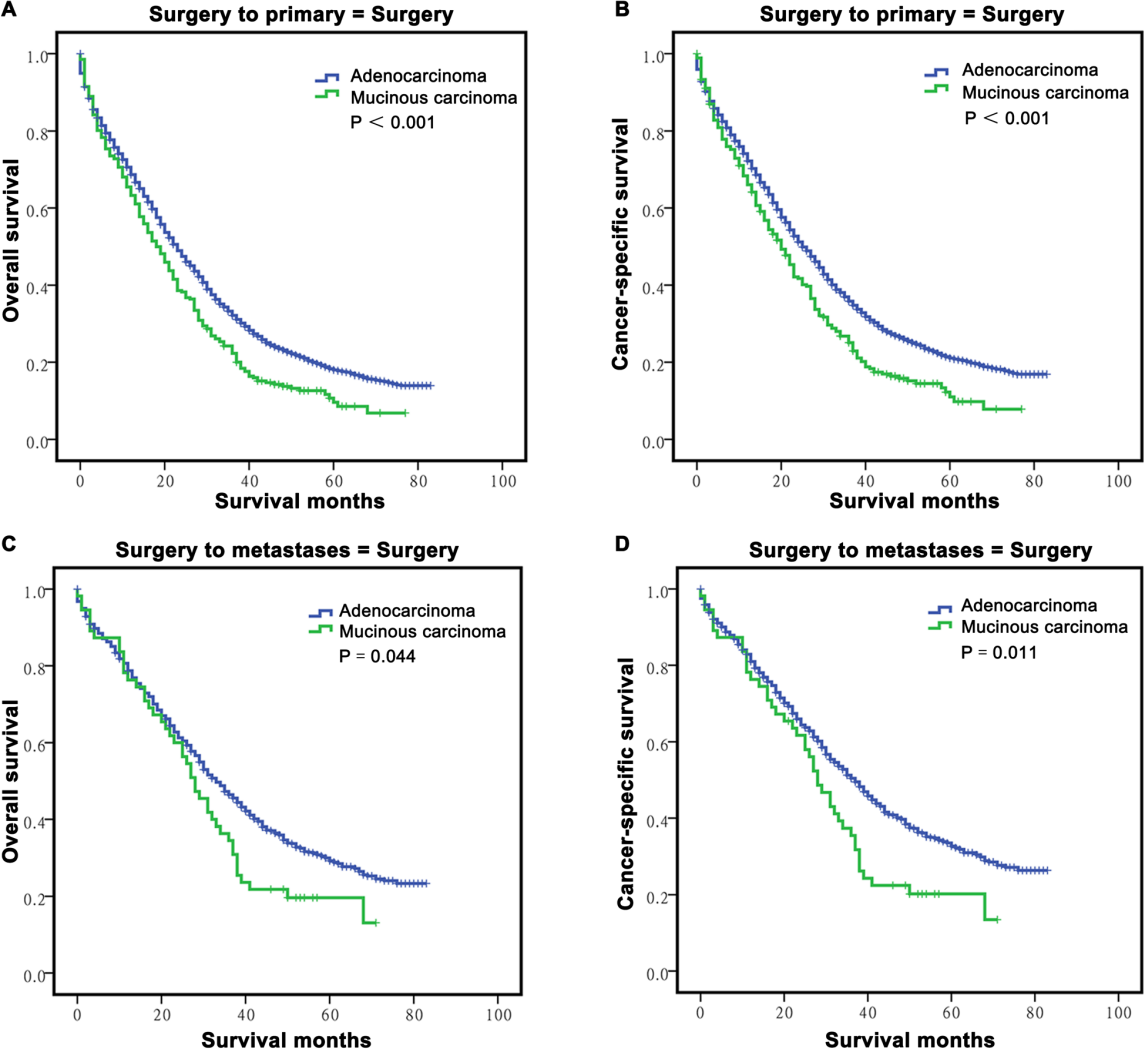
The stratified analysis for long-term survival of CLM according to resection of tumor lesions. A-B: The survival curves showed that the M-CLM group had poorer OS **a** and CSS **b** than the A-CLM group in patients with surgery to primary lesion; **c-d**: The survival curves showed that the M-CLM group had poorer OS **c** and CSS **d** than the A-CLM group in patients with surgery to metastatic lesion

Then, we continued to explore the survival differences via stratification of surgery into primary or metastatic lesion resection. The results showed that among patients who underwent surgery for primary lesion resection, M-CLM patients had poorer OS and CSS than A-CLM patients (P *P* < 0.05 each, Fig. 4a-b). Among patients who underwent surgery for metastatic lesion resection, M-CLM patients also had poorer OS and CSS than A-CLM patients (*P* = 0.044 and *P* = 0.011, respectively, Fig. 4c-d).

### Effect of surgical option for the primary lesion on survival in M-CLM

**Fig. 5:**
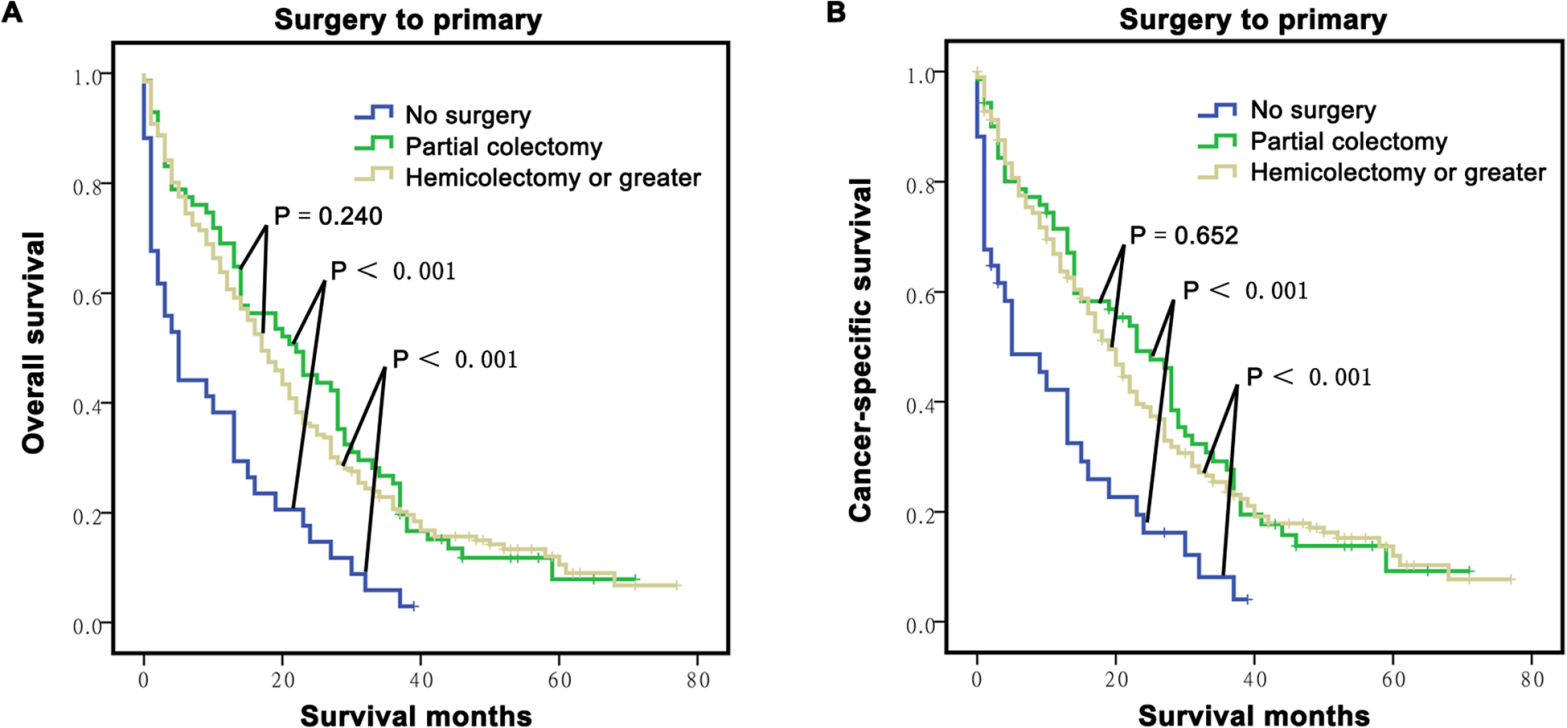
Long-term survival of M-CLM according to surgery type of primary lesion resection. **a**: The survival curves showed that the M-CLM patients who accepted partial colectomy had the similar OS compared with those group who accepted hemicolectomy or greater, but better than those didn’t accept surgery. **b**: The CSS analysis also showed the similar results

There is also controversy regarding the selection of surgical option for the primary lesion in CLM to date; thus, we further analysed the surgical options in terms of survival in M-CLM. A total of 272 (88.89%, 272/306) M-CLM patients underwent surgery in this study, partial colectomy (26.10%, 71/272) and hemicolectomy or more extensive colectomy (72.06%, 196/272) were the most common options. The results showed that partial colectomy had a similar OS to hemicolectomy or more extensive colectomy (*P* = 0.240) but better OS than the no surgery group (*P* < 0.001, Fig. 5a). The CSS analyses also showed similar results (Fig. 5b).

### Prognostic risk factors for survival in M-CLM

Survival for M-CLM is poor, and we need to explore the potential prognostic risk factors for survival for this condition. We analysed the risk factors for OS and CSS of M-CLM by univariable and multivariable Cox proportional hazards regression models in this study. The univariable analysis results showed that black race, pT3–4 stage and surgery for either or both lesions (Either lesion HR = 0.506, 95% CI: 0.349–0.734; both lesions HR = 0.314, 95% CI: 0.198–0.497) were associated with better OS in M-CLM (*P* < 0.05 for all, Table 2). Black race, pT3–4 stage, and surgery for either or both lesions were also associated with better CSS in M-CLM (*P* < 0.05 for all, Table 2). The multivariable analysis demonstrated that only surgery type was an independent prognostic factor for better OS, and black race, pT3–4 stage and surgery type were associated with better CSS in M-CLM (P < 0.05, Table 3). .

**Table 2:** Univariate analysis of factors associated with overall survival and cancer-specific survival of M-CLM

Variable	OS		CSS	
	HR(95%CI)	***P***	HR(95%CI)	***P***
**Race**				
White	1	0.064	1	0.025
Black	0.735 (0.543–0.996)	0.047	0.691 (0.506–0.944)	0.020
Others	0.562 (0.317–0.999)	0.050	0.502 (0.273–0.924)	0.027
**Age (years)**				
≤ 60	1		1	
>60	1.128 (0.876–1.453)	0.349	1.066 (0.821–1.385)	0.629
**Sex**				
Female	1		1	
Male	0.886 (0.698–1.124)	0.319	0.888 (0.693–1.139)	0.350
**CEA**				
Normal	1	0.429	1	0.281
Elevated	1.195 (0.800–1.784)	0.385	1.379 (0.890–2.138)	0.151
Unknown	1.324 (0.861–2.035)	0.202	1.456 (0.911–2.328)	0.117
**Size (cm)**				
≤ 5	1	0.153	1	0.192
>5	1.189 (0.922–1.533)	0.182	1.157 (0.888–1.508)	0.281
Unknown	1.455 (0.964–2.197)	0.074	1.463 (0.955–2.241)	0.081
**Tumor number**				
Solitary	1		1	
Multiple	1.244 (0.921–1.681)	0.155	1.073 (0.772–1.492)	0.675
**Location**				
Right colon	1	0.996	1	0.939
Transverse colon	1.003 (0.675–1.492)	0.987	1.038 (0.687–1.567)	0.859
Left colon	0.989 (0.759–1.290)	0.937	1.049 (0.797–1.380)	0.734
**Differentiation**				
Grade I/II	1	0.528	1	0.547
Grade III/IV	1.000 (0.754–1.326)	0.998	1.013 (0.755–1.358)	0.933
Unknown	1.221 (0.855–1.745)	0.271	1.229 (0.847–1.783)	0.278
**pT stage**				
**0–2**	1	0.004	1	0.002
**3–4**	0.603 (0.392–0.929)	0.022	0.570 (0.367–0.888)	0.013
Unknown	1.104 (0.610–1.997)	0.745	1.105 (0.602–2.028)	0.747
**pN stage**				
**N0**	1	0.117	1	0.192
**N+**	0.844 (0.629–1.134)	0.261	0.892 (0.652–1.219)	0.473
Unknown	1.502 (0.787–2.867)	0.217	1.570 (0.796–3.097)	0.193
**Surgery type**				
No surgery	1	< 0.001	1	< 0.001
Surgery to primary or metastatic lesion	0.506 (0.349–0.734)	< 0.001	0.497 (0.337–0.735)	< 0.001
Both	0.314 (0.198–0.497)	< 0.001	0.330 (0.205–0.531)	< 0.001
Others	1.080 (0.259–4.509)	0.916	1.201 (0.286–5.037)	0.802

**Table 3:** Multivariable analysis of factors associated with OS and CSS of M-CLM

Variable	OS		CSS	
	HR(95%CI)	***P***	HR(95%CI)	***P***
**Race**				
White		NS	1	0.008
Black			0.701 (0.499–0.986)	0.041
Others			0.362 (0.183–0.715)	0.003
**Surgery type**				
No surgery	1	0.004	1	0.017
Surgery to primary or metastatic lesion	0.478 (0.265–0.862)	0.014	0.497 (0.267–0.924)	0.027
Both	0.316 (0.163–0.609)	0.001	0.350 (0.176–0.696)	0.003
Others	1.080 (0.182–4.159)	0.862	0.864 (0.177–4.218)	0.856
**pT stage**				
**0–2**		NS	1	0.039
**3–4**			0.513 (0.306–0.860)	0.011
Unknown			0.785 (0.365–1.688)	0.535

## Discussion

Surgery for colon cancer with liver metastasis is a critical and controversial issue that continues to be discussed to this day. Although most researchers believe that completed resection of both the primary and metastatic lesions would provide a survival advantage over systemic therapy, the main dispute is whether palliative resection of some lesions would be beneficial for patients, especially resection only for the primary colon cancer or for the liver metastasis [26]. Moreover, systemic chemotherapy, molecular targeted therapy, immunotherapy, portal vein or hepatic artery embolization and radiofrequency ablation have been playing an increasingly more important role in mCC treatment and might provide a potentially longer survival and tumour downstaging [5, 22, 26, 27]. This situation has resulted in surgery being less frequently used as treatment for CLM, and many studies support the view that surgery would bring more trauma, stress and immunosuppression for CLM patients than other treatments, probably prompt tumour growth, and recurrence and would not bring survival benefits [23, 28–32]. However, some studies clearly state that resection of the primary colon cancer or liver metastasis is associated with improved survival, and suggested a more aggressive method for treating incurable diseases [22, 33–35].

This dilemma is amplified in M-CLM, because MC is always characterized by peritoneal implantation and metastases at multiple sites which increase the difficulty of completed resection [8, 19, 36]. Moreover, most studies consider MC histology to be an adverse prognostic factor for survival, as well as that of M-CLM, increasing the concerns regarding surgery [10, 15, 17]. However, the relatively low response to systemic therapy in MC compared with that in AC has caused a rethinking of surgery for M-CLM [15, 36]. In this study, we found that M-CLM also had similar general features to MC, such as greater right colon localization, larger tumour size and more advanced pT and pN stages than A-CLM, but the long-term survival of overall M-CLM and A-CLM overall were comparable. This overturns the traditional thinking that MC hasd poorer survival than AC, especially when diagnosed at a high stage (III/IV) [14, 37]. However, our findings are consistent with some recent studies indicating that survival in all stages of MC was poorer than that in AC, but stage IV MC had similar survival as AC [17, 38]. These findings indicated that although M-CLM had specific clinicopathological features, the long-term survival is comparable with that of A-CLM.

Another important finding of the present study was that regardless of whether surgery was performed for both the primary and metastatic lesions or for only one of the lesions for CLM patients, the survival was better than that for no surgery. This conclusion was also verified by stratification of M-CLM and A-CLM by surgery type and confirmed the importance of surgery for survival benefits for M-CLM, which has also been supported by previous studies [33, 35]. We also explored the potential independent risk factors for survival in M-CLM by univariable and multivariable analyses. The results also showed that surgery plays a dominant role in improving OS and CSS, regardless of whether surgery was performed for both the primary and metastatic lesions or for either of the lesions. These results once again highlighted the importance of surgery for improving the prognosis of M-CLM. However, we further found that M-CLM had poorer OS and CSS than A-CLM in the group of patients who underwent any surgery. This finding was different from studies on surgery for stage IV MC [17, 39] but similar to a recent study from Italy that found that M-CLM was associated with worse OS and disease-free survival [8]. One potential explanation for the discrepancy is that the studies on stage IV MC did not stratify the sub-types of M-CLM, since M-CLM is always accompanied by metastasis in other sites and/or the peritoneum, which would worsen the prognosis [8, 15, 36]. Another possible reason is that adjuvant chemotherapy is an important option for postoperative treatment for M-CLM, although this study did not include this information. However, M-CLM is always resistant to systemic chemotherapy, which might also lead to relatively poor survival after surgery [15, 40].

The type of surgery for the primary lesion is also the most debated issue for M-CLM and commonly include partial colectomy and hemicolectomy or more extensive colectomy. Some surgeons tend to choose partial colectomy because M-CLM is a terminal stage disease and surgery cannot improve survival or may even result in a poorer prognosis [28–30]. However, others believe that extended resection, such as hemicolectomy or more extensive colectomy, would provide the chance for subsequent curable resection or greater sensitivity to systemic chemotherapy, which might prolong survival [31, 34, 35]. In the present study, we found that partial colectomy provided a similar OS and CSS to hemicolectomy or more extensive colectomy. This finding strengthened the concept of minimizing trauma for advanced cancer. There are some potential speculations for this, but it is most likely that extended resection may damage the immune system and homeostasis and sometimes even promote tumour growth and metastasis [23]. Thus, the appropriate surgery option should be selected carefully when an operation decision is made for M-CLM.

This study identified the important role of surgery for improving survival in M-CLM. However, there were also some limitations in the study. First and foremost, we could not obtain pre- and/or post-operative systemic therapy information, which could weaken the scientific and academic rigour of the manuscript. Second, this study could not determine whether patients received primary and metastatic lesion resection synchronously or separately. Third, our study enrolled patients with pathological confirmation and detailed staging information in the SEER database, which would exclude many metastatic disease patients without a pathological diagnosis. Thus, more well-designed retrospective and prospective multi-centre studies are needed in the future to overcome these weaknesses.

## Conclusion

In conclusion, this study identified that M-CLM had distinct clinicopathological characteristics from A-CLM and highlighted that surgery could improve long-term survival and is an independent, favourable prognostic factor for survival regardless of whether either or both lesions were resected. In addition, partial colectomy might be a non-inferiority selection for M-CLM treatment according to the results from this study. In conclusion, our study updated the understanding of surgery for MC with colon carcinoma metastasis.

## Supplementary information

**Additional file 1 Table S1.** The demographic and clinicopathological features of mucinous colon adenocarcinoma liver metastasis (M-CLM) and classical colon adenocarcinoma liver metastasis (A-CLM) patients after Propensity Score Match (PSM).

**Additional file 2 Figure S1.** Long-term survival of CLM patients after PSM. A-B: The survival curves showed that the overall M-CLM group had similar overall survival (OS) (A) and cancer-specific survival (CSS) (B) with the A-CLM group after PSM; C-D: The survival curves showed that the M-CLM group received any surgery had poorer OS (C) and CSS (D) than the A-CLM group after PSM; E-F: the survival curves showed M-CLM and A-CLM groups had similar OS (E) and CSS (F) when didn’t perform any surgery after PSM.

## Data Availability

All data generated or analysed during this study are available from the SEER repository (https://seer.cancer.gov/). First, we submitted a data retrive request and be authorized by the SEER data with a username of 16526-Nov2018, then we extracted the eligible data from the SEER database using the SEER program.

## Abbreviations

MC: Mucinous adenocarcinoma
CC: Colon carcinoma
CLM: Colon cancer liver metastases
M-CLM: MAC of CC with liver metastases
SEER: End Results
OS: Overall survival
CSS: Cancer-specific survival
AC: Adenocarcinoma
IBD: Bowel diseases
CEA: Carcinoembryonic antigen
HRs: Hazard ratios
CIs: Confidence intervals
