# Supplementary material for: Surgery improves the prognosis of colon mucinous adenocarcinoma with liver metastases: a SEER-based study
Source: BMC Cancer. 2020 Sep 23;20:908. doi: 10.1186/s12885-020-07400-4 (PMC7510088; doi:10.1186/s12885-020-07400-4)
Supplement: Supplementary file 1 — Additional file 1 Table S1. The demographic and clinicopathological features of mucinous colon adenocarcinoma liver metastasis (M-CLM) and classical colon adenocarcinoma liver metastasis (A-CLM) patients after Propensity Score Match (PSM). [file 12885_2020_7400_MOESM1_ESM.doc]

**Table S1. The demographic and clinicopathological features of mucinous colon adenocarcinoma liver metastasis (M-CLM) and classical colon adenocarcinoma liver metastasis (A-CLM) patients after Propensity Score Match (PSM).**

| **Variables** | **A-CLM (306)** | **M-CLM (306)** | **P value** |
| --- | --- | --- | --- |
| **Race** |  |  |  |
| White | 232(75.8%) | 232(75.8%) | 1 |
| Black | 55(17.7%) | 55(18.0%) |  |
| Others | 19(6.2%) | 19(6.2%) | 1 |
| **Age (years)** |  |  |  |
| ≤ 60 | 101(33.0%) | 101(33.0%) |  |
| ＞60 | 205(67.0%) | 205(67.0%) | 1 |
| **Sex** |  |  |  |
| Female | 153(50.0%) | 153(50.0%) |  |
| Male | 153(50.0%) | 153(50.0%) | 1 |
| **CEA** |  |  |  |
| Normal | 30(9.8%) | 33(10.8%) |  |
| Elevated | 174(56.9%) | 184(60.1%) |  |
| Unknown | 102(33.3%) | 89(29.1%) | 0.520 |
| **Size (cm)** |  |  |  |
| ≤5 | 143(46.7%) | 119(38.9%) |  |
| ＞5 | 94(30.7%) | 156(51.0%) |  |
| Unknown | 69(22.5%) | 31(10.1%) | <0.001 |
| **Tumor number** |  |  |  |
| Solitary | 248(81.0%) | 248(81.0%) |  |
| Multiple | 58(19.0%) | 58(19.0%) | 0.541 |
| **Location** |  |  |  |
| Right colon | 140(45.8%) | 181(59.2%) |  |
| Transverse colon | 32(10.5%) | 34(11.1%) |  |
| Left colon | 134(43.8%) | 91(29.7%) | 0.001 |
| **Differentiation** |  |  |  |
| Grade Ⅰ/Ⅱ | 191(62.4%) | 189(61.4%) |  |
| Grade Ⅲ/Ⅳ | 70(22.9%) | 79(25.8%) |  |
| Unknown | 45(14.7%) | 39(12.7%) | 0.608 |
| **pT stage** |  |  |  |
| **0-2** | 29(9.5%) | 25(8.2%) |  |
| **3-4** | 215(70.3%) | 259(84.6%) |  |
| Unknown | 62(20.3%) | 22(7.2%) | <0.001 |
| **pN stage** |  |  |  |
| **N0** | 95(31.0%) | 62(20.3%) |  |
| **N+** | 184(60.2%) | 233(76.1%) |  |
| Unknown | 27(8.8%) | 11(3.6%) | <0.001 |
| **Examined lymph nodes** | 12.74±11.96 | 16.26±10.41 | <0.001 |
| **Positive lymph nodes** | 4.21±5.31 | 5.37±5.84 | 0.025 |
| **Surgery type** |  |  |  |
| No surgery | 86(28.1%) | 34(11.1%) |  |
| Any surgery | 220(71.9%) | 272(88.9%) | <0.001 |
