# Supplementary figures and images for: Surgery improves the prognosis of colon mucinous adenocarcinoma with liver metastases: a SEER-based study
Source: BMC Cancer. 2020 Sep 23;20:908. doi: 10.1186/s12885-020-07400-4 (PMC7510088; doi:10.1186/s12885-020-07400-4)

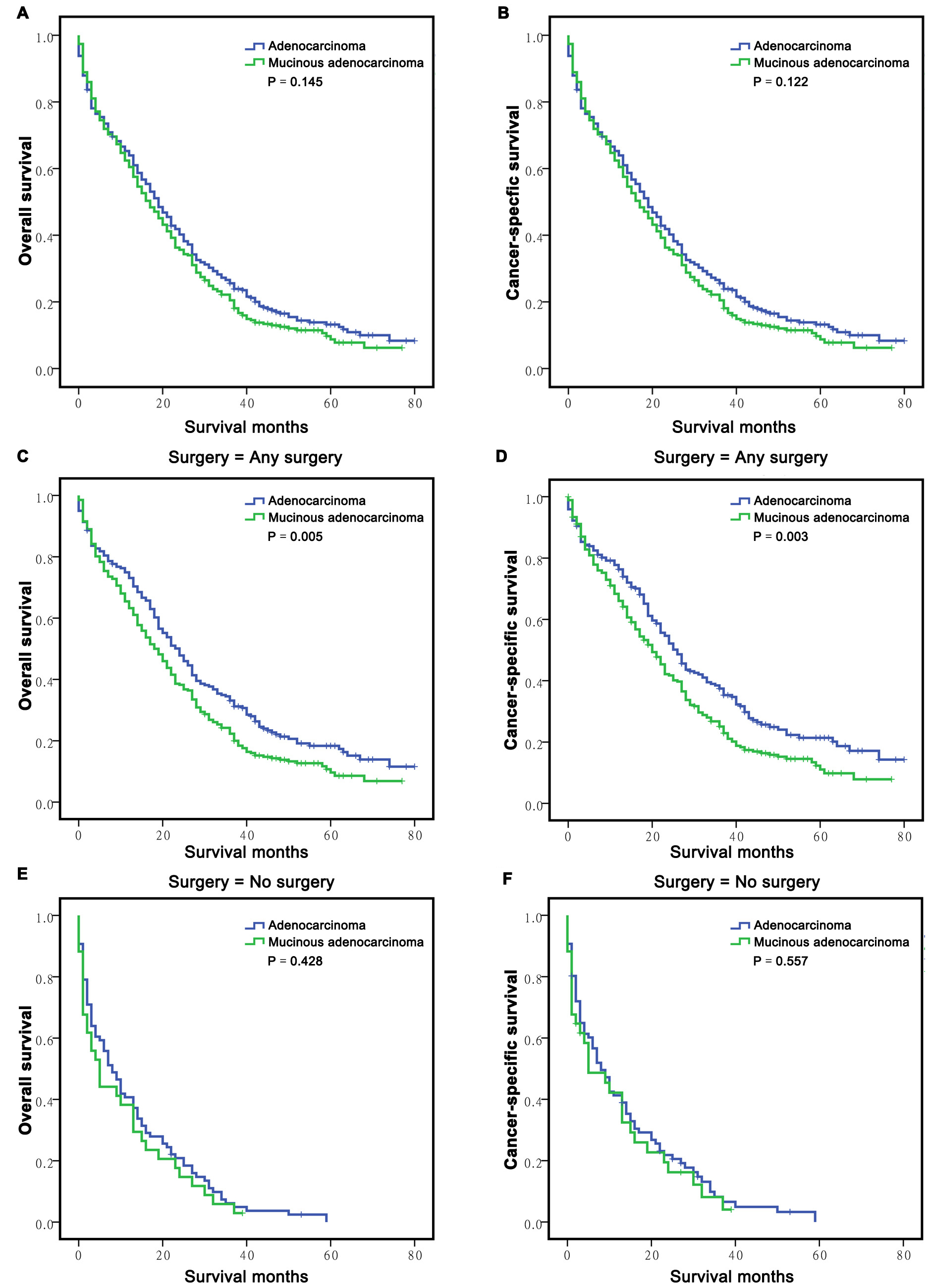

Supplement: Supplementary file 2 — Additional file 2 Figure S1. Long-term survival of CLM patients after PSM. A-B: The survival curves showed that the overall M-CLM group had similar overall survival (OS) (A) and cancer-specific survival (CSS) (B) with the A-CLM group after PSM; C-D: The survival curves showed that the M-CLM group received any surgery had poorer OS (C) and CSS (D) than the A-CLM group after PSM; E-F: the survival curves showed M-CLM and A-CLM groups had similar OS (E) and CSS (F) when didn’t perform any surgery after PSM. [file 12885_2020_7400_MOESM2_ESM.zip › Figure S1R3.jpg]
